# Dicing with data: the risks, benefits, tensions and tech of health data in the iToBoS project

**DOI:** 10.3389/fdgth.2024.1272709

**Published:** 2024-01-31

**Authors:** Niamh Aspell, Abigail Goldsteen, Robin Renwick

**Affiliations:** ^1^ Innovation & Research, Trilateral Research Ltd., Waterford, Ireland; ^2^ Data Security and Privacy, IBM Research, Haifa, Israel

**Keywords:** privacy, healthcare, ethics, machine learning, data protection, trust

## Abstract

This paper will discuss the European funded iToBoS project, tasked by the European Commission to develop an AI diagnostic platform for the early detection of skin melanoma. The paper will outline the project, provide an overview of the data being processed, describe the impact assessment processes, and explain the AI privacy risk mitigation methods being deployed. Following this, the paper will offer a brief discussion of some of the more complex aspects: (1) the relatively low population clinical trial study cohort, which poses risks associated with data distinguishability and the masking ability of the applied anonymisation tools, (2) the project's ability to obtain informed consent from the study cohort given the complexity of the technologies, (3) the project's commitment to an open research data strategy and the additional privacy risk mitigations required to protect the multi-modal study data, and (4) the ability of the project to adequately explain the outputs of the algorithmic components to a broad range of stakeholders. The paper will discuss how the complexities have caused tension which are reflective of wider tensions in the health domain. A project level solution includes collaboration with a melanoma patient network, as an avenue for fair and representative qualification of risks and benefits with the patient stakeholder group. However, it is unclear how scalable this process is given the relentless pursuit of innovation within the health domain, accentuated by the continued proliferation of artificial intelligence, open data strategies, and the integration of multi-modal data sets inclusive of genomics.

## 1 Introduction

Balancing the risks and benefits of using medical and genomics data for diagnostic clinical decision support tools is a complex task. Principles of medical ethics such as autonomy, beneficence, and non-maleficence are weighed against broader concepts such as privacy, security, safety, bias, explainability, and cost. Concerns are further compounded by the proliferation of Artificial Intelligence (AI) in the health domain, intending to improve healthcare by aiding the clinician's knowledge or by highlighting suspicious observations that are otherwise unobservable. In addition to fundamental societal harm, careless deployment of AI technologies may result in negative brand reputation, lawsuits, and regulatory fines. This has led to the rise of the concept of Trustworthy AI, sometimes called Responsible AI or AI Ethics^1^. Making AI systems trustworthy depends on the ability to ensure that they are fair, robust, explainable, accountable, respectful of the privacy of individuals and cause no harm. Trustworthy or Responsible AI typically entails considering these aspects when designing, implementing, and deploying AI-based solutions.

This paper will discuss the iToBoS project, in which an AI diagnostic platform for early detection of melanoma is being developed. Assuring the project's solutions are produced in an ethically and socially responsible manner, with regulatory compliance at their core, is one of the project's primary goals. Stating the goal of the project is relatively straightforward but achieving the goal adequately is less so—especially when research tasks are considered alongside an evolving health sector (1). This paper will communicate existing tensions in the development of the iToBoS tools, with specific focus on the privacy aspect, which is one of the main trustworthiness aspects tackled in the project. We will outline the AI Privacy technologies that are deployed as risk mitigation measures. This includes tools for anonymising the AI model training data and AI models themselves, and to support adherence to the data minimisation principle. The article will conclude with a brief discussion on the existing complexities of balancing risk and benefit when developing AI diagnostic platforms, with specific focus on understanding perspectives of privacy, explainable AI, and the cost/benefit calculation from predominant stakeholders such as patients, clinicians, and the wider health research community.

## 2 iToBos and its data

IToBoS is a European-funded research project, in which the core research task is to develop an AI diagnostic platform for the early detection of melanoma. The platform includes a novel total body scanner and a Computer-Aided Diagnostics (CAD) tool, incorporating relevant data such as patients' clinical data, phenotypic data, genetic data, skin imaging, and records of familial melanoma. The AI component of the platform has two primary functions. First, high-resolution skin images will be captured, analysed and classified to aid melanoma detection and classification. Secondly, the images will be integrated with available patient data to train machine learning (ML) models in the development of an AI-based “cognitive assistant” (AICA). The iToBoS platform will subsequently provide clinicians with a personalised risk assessment to support the early diagnosis of melanoma. The intention is to improve the skin melanoma detection and classification processes (previously a labour-intensive task completed manually by clinicians), and to provide further insights into patient health through the detection of patterns across otherwise indirectly connected data sets.

With the direct involvement of clinicians in the project, iToBoS was able to select a range of features to include in the development of the AICA. The data points have demonstrated, through prior melanoma research, relevance to skin melanoma prognosis (a prediction of the probable course and outcome of a disease). These include data pertaining to the patient's phenotype such as skin pigmentation, ancestry, hair and eye colour, and lifestyle factors, such as sun exposure habits (2, 3). In addition to these recognised phenotypic determinants, individuals with certain hereditary gene mutations also have an increased, or compounded, risk of developing melanoma (4). The collection of phenotypic and genetic data has raised concerns in recent years, as they have been targeted for exploitation by researchers, employers, insurers, and law enforcement (5). Genomic studies have identified various susceptibility variants for melanoma. This means that researchers have identified genomic variants that seem to determine an individual's susceptibility to developing melanoma. Combining these variants into polygenic risk scores (PRS) may offer important information to clinicians and provide an additional layer of privacy (6). The risk scores are used to estimate patients' risk of developing particular diseases. In the iToBoS project clinicians will evaluate and assign a PRS to patients who “opt-in” for genetic screening.

## 3 Privacy impact assessment+

In iToBoS, project specific concerns related to medical data and genomics are evaluated through the conducting of a Privacy Impact Assessment + (PIA+). The process considers privacy from the standpoint of the current ISO PIA standard (ISO/IEC 29134:2017) (7). In the project context, the “+” designates that additional domains are also considered alongside privacy, such as ethics, society, and law. At a high level, the PIA + tool is a vehicle for identifying possible risks, forecasting implications, and proposing mitigation measures during the development lifecycle. It has been used effectively across a number of recently funded EU projects (e.g., SOTER^2^, EUNOMIA^3^, and AQUA3S^4^). Additionally, the PIA + process is completed in a public and open manner, acting as a vehicle for building trust, as well as an accountability and transparency tool (8, 9).

It is intended to:

• Help minimize potential risks and harms, while signposting future (post-project) concerns for the iToBoS technology.
• Support the pursuit of compliance with regulatory frameworks, such as the European General Data Protection Regulation (GDPR) (10).
• Contribute to informed decision-making and development of mitigation measures to minimise privacy, social and ethical risks for individuals, organisations, and society.

In practice, the PIA + is conducted in a similar manner to a risk assessment. System features, assets and data flows are initially identified, with collaborative analysis then conducted to understand system specific vulnerabilities and their associated risks. These risks are defined qualitatively, with a description communicated alongside a qualification of the potential impact (i.e., low, medium, high), and probability of occurrence (i.e., low, medium, high). The process is analytical in nature, as opposed to empirical, but is used to focus efforts across the development team, and drive ideation and creation of solutions for identified risks.

## 4 AI privacy

One of the core elements of iToBoS is the development of a privacy-respecting AICA. In order to develop this, a number of tools are deployed to ensure that any data used during the AI development process, as well as the resulting models, are adequately protected. In an AI system, it is necessary to ensure that data (whether for testing, validation or training) is adequately and lawfully collected, stored, protected, and governed. It is also critical that there is a legitimate purpose for processing. Recent studies have shown that a malicious third party with access to a trained machine learning (ML) model, even without access to the training data itself, can reveal sensitive information about the people whose data was used to train the model (11). It is therefore important to address privacy aspects both with the datasets and resulting models.

The technical approaches taken to address AI privacy risks in the iToBoS project will be described, including anonymising training data to yield an anonymised model, and applying data minimisation to the newly collected data for analysis.

Both AI privacy methods applied to iToBoS are currently available in the open-source ai-privacy-toolkit (12). Initial results indicating the applicability of these technologies to health-related data have been recently demonstrated (13).

### 4.1 Anonymising models

According to GDPR, anonymous data is data from which the data subject is no longer identifiable. It has been shown in the past that simple removal of direct identifiers is not enough to achieve this goal (14). Therefore, more sophisticated methods such as k-anonymity and differential privacy have been developed. As the iToBoS project intends to publish research datasets and models, it is important to apply one of these techniques, to reduce the risk of patient re-identification in published results.

#### 4.1.1 Possible approaches

K-anonymity (15) is a method that attempts to reduce the probability of people being identified when publishing datasets that contain personal information, even when linking them with other data sources. It involves generalizing some of the attributes, and sometimes also deleting select records, until each record in the dataset is indistinguishable from at least k−1 others. Traditionally, ML models trained on anonymised data tend to suffer from very poor accuracy. Therefore, a model-guided anonymisation method was proposed (16) that utilizes knowledge encoded within the model to create an anonymisation tailored to that specific model, thus retaining more utility than non-tailored approaches.

Differential privacy (DP) is another known approach to reduce the effect of individual data records on a model's outcome (17). This is achieved by adding noise during training. This type of approach requires changing the ML algorithm implementation and is therefore more difficult to use in practice. Yet another possible approach entails generating synthetic data that shares desired characteristics with the original data (18).

The iToBoS project intends to publish training datasets as part of iToBoS challenges. These are open hackathon type events where development teams can experiment with novel data sets—similar to the International Skin Imaging Classification Challenges (ISIC) (19). The project will also likely release the models themselves, so a model-guided anonymisation approach (16) that enables anonymising tabular data and models in the same manner, whilst providing adequate privacy protection guarantees was selected.

Typically, k-anonymity methods require that a list of quasi-identifiers (QI) be determined. These are attributes (features) that may be used to re-identify individuals when combined with each other or linked with other external datasets. To determine which features should be treated as QI in the tabular data collected in iToBoS, we plan to both use as reference the list of HIPAA identifiers^5^ and apply a risk analysis tool (20) to identify potential QIs.

### 4.2 Minimising the collected data

GDPR dictates the principle of *data minimisation* which requires organisations to collect only the data that is required to achieve a given purpose. Advanced ML algorithms, such as deep neural networks, tend to consume large amounts of data to produce a prediction, and often result in “black box” models where it is difficult to derive exactly which data influenced the decision (21).

To this end, a method for data minimisation that can reduce the amount and granularity of input data used to perform predictions by ML models was developed (22). Once a model is trained and validated, the method allows a re-evaluation of exactly what data is required for the model to be accurate. Using knowledge encoded in the model, it tries to determine whether input features may be generalized, or completely removed, without reducing overall model accuracy. For example, instead of exact ages, it may be possible to use 5- or 10-year ranges.

Even if there are cases where all the collected data is required to achieve the model's original accuracy and no generalisation may be performed, it still must be demonstrated that this is the case.

## 5 Risk v utility

As mentioned, training ML models with sensitive and personal data poses enhanced privacy risks. Once algorithms have been trained, an adversary observing the model but without access to the training data, can apply inference algorithms to re-identify information related to the training cohort (23). Reports published by the Information Commissioner Office (ICO) and the National Institute of Standards and Technology (NIST) highlight the privacy risks of data from ML models, and how the risks of using the AI tools should be outweighed by its utility (24, 25). A proposed response has emerged in the form of guidance, authored by cybersecurity researchers and focused on the development of privacy risk evaluation tools (26). The application of probabilistic programming to quantify indirect data leakage using tools such as “Privug” offer solutions for both privacy researchers, and data controllers, to conduct analysis in order to make informed decisions when anonymising data (27, 28). In a similar fashion, a recent publication from the European Union Agency for Cybersecurity details risks associated with medical imaging data for diagnosis (29). The agency outlines 29 measures (as well as associated threats and vulnerabilities), split into generic and specific controls.

While tools and methods have emerged in response to the identified risks, the task for AI developers, research teams, and the health domain remains complex. Proposed mitigations include tasks such as regular auditing, bias detection and mitigation strategies, AI conformity assessments, and ongoing compliance with data protection obligations. Privacy- and Security-by-Design strategies are recommended, as well as formal Data Protection- or Privacy- impact assessment processes. These methods are viewed as integral components of responsible design, development, and deployment. However, even given the array of risk mitigation methods available and recommended, complexities remain. It is rational to assume that no overarching panacea to emerging health domain risks exists, especially as risks are continually spurred on by relentless adoption of new technology. It is also rational to assume that the application of formal mitigation strategies slows the pursuit of progress, creating burdens (both technical and practical) for compliance managers, data ethicists, ethics managers, impact assessors, computer scientists, and so on. In the section below we will outline some of the complexities found within the iToBoS project, and demonstrate how these might be viewed as representative of wider complexities found in the health domain.

## 6 iToBos complexities

Within iToBoS, tasks primarily focused on risk and impact mitigation are included but delivering them adequately has posed problems. Firstly, some unique challenges arise when applying anonymisation to data collected in iToBoS. The most predominant issue of concern is the relatively small size of the dataset. The initial study planned to collect data from around 500 patients. This means that in order to gain meaningful insights, the selected privacy parameter (*k*-value) cannot be too high. A related issue stems from the sparseness of some of the features included in the data set. For example, since only a few clinical sites are involved, country of residence tends to be very centralised to the country where the study is being conducted, with only a few outliers. Country of birth is also similarly distributed, with a very high tendency (>80%) towards the site country, and very sparse presence in other locations. This may be solved by manually removing some of the features or records or by binning multiple possible feature values together, before starting the automatic anonymisation process.

Secondly, data collection is dependent on adequate informed consent being collected from patients. In practice, this means that patients are required to fully understand how data is being processed, by whom, the purpose for processing, and what the initially identified risks are. Adequately explaining how machine learning algorithms will be deployed, what inferences they may make, and what patterns they may detect while ingesting multi-modal data sets, however, is not simple. Prior research has identified problems with clinical trial consent (30), and this is further complicated in iToBoS given the specific masking techniques being applied to multi-modal collected data. What level of understanding do we expect patients to have of machine learning technologies, and how cognisant can we realistically expect them to be of the broader risks as well as the proposed mitigation strategies provided by the anonymisation tools?

Third, while iToBoS utilises project specific clinical data for the development of the AICA, the project also commits to contributing to the ISIC challenges and associated archive (19). The ISIC archive is a platform for open and collaborative AI-based skin melanoma diagnosis and promotes the sharing of clinical skin imaging data for the benefit of researchers, patients, clinicians, and the wider health research community. This open data commitment poses additional risks for the study cohort, and so demands that additional anonymisation efforts are applied to the collected data. While specific methods may be adequate to mitigate privacy concerns at a local level, additional steps are required to sufficiently mitigate risks if data is intended to be shared for further processing. This is especially complex given the proposed release of multi-modal data sets, which may afford a greater degree of inference given possible data combinations.

Lastly, clinicians currently have limited understanding of how machine learning algorithms infer specific prognoses for melanoma. This limitation affects clinicians’ ability to adequately explain to the patient how the AICA reached its conclusion. The black-box nature of algorithms has the potential to alter aspects of classical medical ethics (31) including accountability, liability, and the ability of the clinician to develop experience and expertise in manual prognosis (and diagnosis) according to professional norms. In the medium to long term, clinicians might become more dependent on the output of a technology, rather than building their own professional corpus on how to compare, contrast, and correlate multi-modal streams of health data. iToBoS has specific explainable-AI (xAI) tasks that seek to mitigate this explainability risk—but formally understanding the xAI requirements has also proven to be problematic. The project will provide an xAI framework so that computer scientists can understand how algorithms have arrived at lesion detection, classification, or overall skin melanoma risk-profile conclusions. However, researchers are also attempting to clarify exactly what sort of information (and in what detail) is required so that clinicians and patients can also understand (and be able to explain) how the AICA has arrived at a specific prognosis, or patient risk score. These two types of explanations differ substantially, and it has proven difficult to balance the two, sometimes competing, requirements. Additionally, the project is continually attempting to balance requirements for privacy, data utility and explainability. Researchers are simultaneously striving for privacy-preserving data ingestion as well as for trained model accuracy and efficiency. Balancing competing requirements is complex and requires problem framing through varied privacy, machine learning, and security lenses (32)—which inevitably slows progress and stifles aspects of innovation. There are tools that can help this calculation, but they inevitably rely on some level of qualitative assessment, based on subjective experience, expertise, and problem framing. While no subjective assessment is perfect, the iToBoS project does try to include multiple stakeholders in the assessment process, with the intended goal to reach some form of broad consensus regarding risks and benefits.

### 6.1 Patient led mitigation

One of the research partners in the iToBoS project is the Melanoma Patient Network of Europe (MPNE). They are a network organisation that includes melanoma patients, carers and advocates drawn from across Europe. Their mission is to provide a platform for communication and collaboration between patients, researchers, and health service providers. They also provide a channel through which initially identified risks can be validated, and mitigation methods developed, in a collaborative fashion—regardless of whether they were identified through qualitative impact assessment processes or formal quantitative assessment of anonymised data. This process allows technologists, model developers, and researchers to understand their role and responsibilities alongside the voice of the patient, as opposed to the vacuum of the computer science laboratory. Researchers can canvas opinions on a wide range of topics, from artificial intelligence to big data, from genomic screening and risk-scoring to ergonomic and inclusive design of skin imaging hardware. This collaboration does not guarantee a perfect outcome, but it helps to foster a more patient-centric project, and allows researchers to understand exactly how well explainability, trustworthiness and privacy mitigations are being perceived and understood by patients, which in turn informs how deeply clinicians might adopt (and trust) algorithmically led decision support systems.

This patient-led strategy is not new, with recent studies being conducted in a wide range of health sub-domains, from the use and adoption of Electronic Health Records (EHR) (33) to machine learning and artificial intelligence (34, 35). Attempting to understand patient views, both positive and negative, allows researchers to frame wider implications and potential apprehensions of emerging technology. It also supports a robust qualitative avenue of enquiry for the risk vs. utility calculation. AI and data privacy remains a high-agenda topic across European and Global policy and regulatory initiatives, but less is known about how patients view AI clinical decision support tools, the associated privacy risks, and the degree to which patients would be willing to share their health data if provided the autonomy to consider the risks and potential health opportunities accurately (36). This sentiment is shared by McDougall (2019), who proposes the need for “value-flexible AI”, essentially moving from clinician-based support tools to shared decision supports, ultimately advocating for continual patient engagement in medical decision making (37). While there is merit in this proposition, it is unclear whether this sort of patient engagement is achievable (or sustainable) in the short to medium term, as AI tools proliferate the market, strongly dictating their adoption into the broader health domain.

## 7 Discussion

Market forces (more often than not) dictate the speed and depth at which new technologies embed themselves into society (38). This is no different in the health domain, even given the complex social, ethical, privacy and security concerns that have (and will continue to be) raised. The strong hand of regulation has been proposed as the predominant risk mitigator, whether enforced through strict obligations regarding the use of AI (39), medical devices (40), or the oversight and regulation of European Health Data Spaces (41). The seeming tension between the European Commission's desire for open-data—governed through its Digital Strategy—and the risks inherent with the generalised sharing of health, genomics, and model data is a concern that is yet to be fully addressed. Information potentially revealed by certain data-led health strategies is classified as sensitive in most (if not all) situations, with risks amplified as machine learning algorithms are applied to a broad range of prognosis and diagnosis methods. However, it is also fair to assume that excessively rigid regulations may limit innovation (42, 43, 44), and potentially restrict society from reaping the full public health benefit, especially when genomics are involved. While the perspective of how exactly market forces skew the evolution of public health has been communicated (45), there is also loose consensus that algorithmic technology can provide immense benefits for societal well-being, bring concrete efficiencies, and provide measurable improvements to the provision of healthcare (46).

Moving forward, conversations should continue to include a multitude of stakeholders—patients, clinicians, advocacy bodies, policy makers, and technologists—but it is still not clear if discussions will provide meaningful resolution to a host of ongoing concerns surrounding explainability, trustworthiness, open-data, privacy, and machine learning. Within iToBoS, efforts have been made to incorporate a wide range of stakeholder views but it is not clear whether these methods are viable at scale. Applying state of the art technologies to iToBoS’ data processing allows project specific controls to be deployed, whilst learnings can also be applied to other health domain use-cases and integrated into high-level policy initiatives. However, it is still not entirely clear how much impact this will have on the wider health domain, given the rapid pace of development we are currently witnessing at the intersection of health, data, and machine learning.

As discussed, the iToBoS project has encountered project specific complexities that can be mitigated, such as issues with the size and distinguishability of the clinical trial cohort data. The project, however, has also encountered broader concerns that it has found more difficult to navigate—especially those surrounding meaningful consent, the required depth and range of algorithmic explainability, and the ongoing commitment to open data sharing. The project consortium has learned that communicating risks and benefits in an inclusive manner is an integral step in facilitating better research practice, as well as providing critical groundwork in establishing public and professional trust in the open data concept. We have also learned the importance of ensuring (and communicating) that proper and correct data protection and privacy technologies have been applied during the research process. Involving patients in this discussion is critical—even if it might seem to slow progress or muddy the risk vs. utility calculation.

Ultimately, it should be remembered that patient groups carry the greatest risk burden and are rewarded with the most potential benefit—regardless of what tool is developed. Integrating their voice throughout the development cycle is the only fair way to assess technologies and gauge whether algorithms have impacted their ability to make fair and proper calculations. Understanding how well patients understand and perceive concepts related to explainability, machine learning data set inference, and multi-modal health data risk-profiling will not solve every nuanced problem, but it will allow us to understand both practical and technical gaps that need bridging, as the health domain continues to evolve.

## Data Availability

The original contributions presented in the study are included in the article, further inquiries can be directed to the corresponding author.

## Footnotes

^1^For a high-level overview of Trustworthy, Responsible or Ethical AI initiatives, the authors point to this reference. https://www.aiethicist.org/frameworks-guidelines-toolkits

^2^ https://cordis.europa.eu/project/id/833923

^3^ https://cordis.europa.eu/project/id/825171

^4^ https://cordis.europa.eu/project/id/832876

^5^ https://www.dhcs.ca.gov/dataandstats/data/Pages/ListofHIPAAIdentifiers.aspx
